# A Review of Catalysts for Hydrogen Production from Methanol

**DOI:** 10.3390/molecules31081345

**Published:** 2026-04-19

**Authors:** Eun Duck Park

**Affiliations:** 1 Department of Energy Systems Research, Ajou University, Suwon 16499, Republic of Korea; edpark@ajou.ac.kr; Tel.: +82-31-219-2384; 2 Department of Chemical Engineering, Ajou University, Suwon 16499, Republic of Korea

**Keywords:** steam reforming, aqueous-phase reforming, methanol dehydrogenation, methanol, hydrogen, catalyst

## Abstract

Methanol is the simplest C1 oxygenated compound possessing the highest hydrogen-to-carbon ratio and can therefore be used as an effective hydrogen carrier. Furthermore, it can be easily transported by land and sea because it is liquid at room temperature and atmospheric pressure. Methanol can be converted into hydrogen via methanol steam reforming (MSR), aqueous-phase reforming of methanol (APRM), or aqueous methanol dehydrogenation (AMDH). In this review, various catalysts for MSR, APRM, and AMDH are summarized. Highly active and stable catalysts that can operate under low steam-to-methanol ratios are needed to increase the economics of the MSR process. Compared with the MSR process, the APRM process is rather simple because the water–gas shift reaction can occur simultaneously; however, more constraints exist in the selection of active metals and supports to ensure high activity and stability under APRM conditions. The inherently low reaction rate compared to MSR and the structural vulnerability of the catalyst under severe hydrothermal conditions are obstacles that the APRM catalysts must overcome. The low intrinsic catalytic activity and the high cost of homogeneous catalysts represent fundamental limitations inherent to AMDH catalysts. Based on a literature survey of MSR, APRM, and AMDH catalysts, some future research directions are also discussed.

## 1. Introduction

Methanol is one of the bulk chemicals with a large market size, with production of about 107 million tons in 2021 [1,2]. Similar to synthesis gas (a mixture of CO and H_2_), it can be converted into various bulk chemicals through established processes, such as methanol-to-gasoline conversion [3], methanol-to-olefin conversion [4], methanol-to-acetic acid conversion [5], etc., in the chemical industry [6,7]. Recently, methanol synthesis utilizing biomass feedstock [8,9] or using CO_2_ and green hydrogen [1,10,11,12,13] has attracted much attention as a means to achieve carbon neutrality.

Methanol possesses the highest hydrogen-to-carbon ratio among C1 oxygenates and remains liquid at room temperature and atmospheric pressure. Consequently, it can be readily transported by land and sea, making it suitable for use as a hydrogen carrier [14,15,16]. Table 1 summarizes some typical reactions to produce hydrogen along with the changes in thermodynamic properties. Methanol and formic acid can be considered as favorable hydrogen carriers because their reactions to produce hydrogen are less thermodynamically limited than others. The enthalpy change for each reaction is also a crucial factor affecting the energy efficiency of this process. Exothermic reactions are preferable to endothermic reactions, and even for endothermic reactions, those with lower reaction enthalpies are more desirable. Consequently, formic acid [17,18], ammonia [18,19,20], and methanol are evaluated as promising hydrogen sources. Among these, methanol may be selected as the optimal candidate when considering hydrogen content and safety concerns.

Methanol steam reforming (MSR) is the typical route for hydrogen production from methanol (entry 2 in Table 1) [21]. It is a gas-phase endothermic reaction, making it more thermodynamically and kinetically feasible at higher temperatures. However, due to the exothermic nature of the water–gas shift (WGS) reaction (entry 3 in Table 1) that accompanies MSR, it is thermodynamically unfavorable to reduce CO concentrations at high temperatures, requiring the use of active catalysts for MSR and WGS at low temperatures.

In order to perform reforming and WGS simultaneously, aqueous-phase reforming (APR) was introduced [22]. The APR of methanol (APRM) is a liquid-phase endothermic reaction, making it more feasible thermodynamically and kinetically at higher temperatures.


(1) $${\mathrm{CH}}_{3}\mathrm{OH}(l)\;+\;H_{2}O(l)\;\to \;{\mathrm{CO}}_{2}(g)\;+\;3H_{2}(g)\;\;\;\;\;\;∆H_{298K}^{o}\;=131\mathrm{kJ}/\mathrm{mol}$$

However, higher pressures are required to maintain the liquid phase at higher temperatures, so a highly active catalyst must be used in the APRM to allow operation at relatively low temperatures and pressures. The advantage of APRM over MSR is that it requires no additional heat to evaporate methanol and water and can produce high-purity, high-pressure hydrogen without any additional WGS process. A thermodynamic and techno-economic performance comparison of APRM and MSR for hydrogen production revealed that APRM reduced variable operating costs through decreased reactant consumption, lowered power demand, and reduced cooling water usage, resulting in lower annual total costs compared to MSR [23]. This advantage of the APRM increases as the concentration of methanol in the feed decreases.

These MSR and APRM processes are based on heterogeneous catalysts. On the other hand, homogeneous organometallic catalysts have been reported to dehydrogenate methanol into H_2_ and CO_2_ under mild conditions [24]. The advantage of this aqueous methanol dehydrogenation (AMDH) process is that it can produce hydrogen at a low operating temperature, below 100 °C, even with a lower water-to-methanol ratio than MSR and APRM.

**Figure 1:**
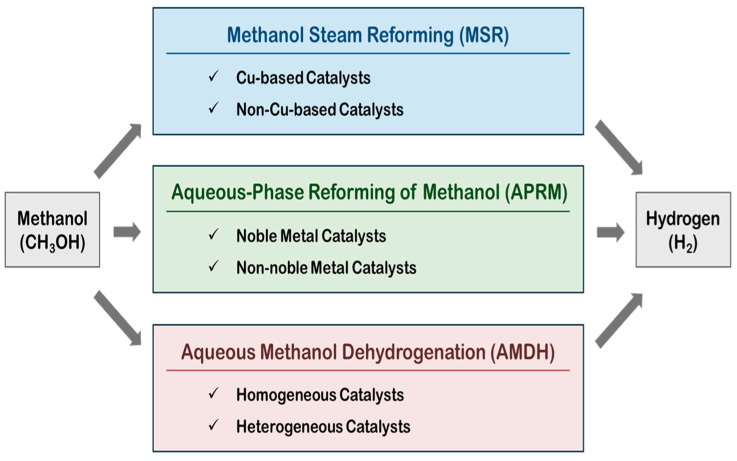
Three representative catalytic routes for hydrogen production from methanol and some typical catalyst systems for each route.

In all these MSR, APRM and AMDH processes, catalysts play a pivotal role in accelerating the conversion rate of methanol and increasing the selectivity to hydrogen (Figure 1). Depending on the target reaction, the appropriate catalyst should be selected. As discussed separately in each section below, several review papers addressing each topic have been reported. However, review papers that comprehensively compare and evaluate each approach in terms of catalytic activity are rare; therefore, a review written from this perspective, reflecting recent research findings, is needed. In this review, catalysts are categorized and summarized according to the reaction. This will be beneficial when selecting or further developing catalysts in related reactions in the future.

## 2. Methanol Steam Reforming (MSR)

Various side reactions can occur during MSR in the presence of a catalyst, as follows.


CH_3_OH(g) ↔ CO(g) +
2H_2_(g) (2)

CH_3_OH(g) ↔ HCHO(g) +
H_2_(g) (3)

CO_2_(g) + H_2_(g) ↔ CO(g) + H_2_O(g) (4)

CO(g) + 3H_2_(g) ↔ CH_4_(g)
+ H_2_O(g) (5)

nCO(g) + (2n + 1)H_2_(g) → C_n_H_2n+2_(g)
+ nH_2_O(g) (6)

nCO(g) + 2nH_2_(g) → C_n_H_2n_(g)
+ nH_2_O(g) (7)

According to the above side reactions, CO, CH_4_, alkanes (C_n_H_2n+2_), and alkenes (C_n_H_2n_) can be produced along with CO_2_ and H_2_. These are undesirable products as they consume hydrogen; therefore, their formation must be minimized by selecting an appropriate catalyst. On the other hand, WGS reaction (entry 3 in Table 1) is desirable as it reduces CO while simultaneously increasing H_2_.

Various catalysts, including Cu-based, Pd-based, and Au-based catalysts, have been reported to be active for MSR [21,25,26,27,28,29,30,31,32,33,34,35,36,37,38,39,40]. Table 2 summarizes some Cu-based catalysts active for MSR [41,42,43,44,45,46,47,48,49,50,51,52,53,54,55,56,57,58,59,60,61,62,63,64,65,66,67,68,69,70,71,72].

Meng et al. [42] stabilized Cu_2_O with amorphous alumina to fabricate a Cu/Cu(Al)O_x_ catalyst adjacent to Cu nanoparticles (entry 2 in Table 2), providing Cu^0^−Cu^+^ active sites, which exhibited a high H_2_ production rate and relatively stable activity. They discovered that key oxygen-containing intermediates (CH_3_O* and HCOO*) adsorbed onto the Cu^0^−Cu^+^ sites with moderate adsorption strength, facilitating electron transfer from the catalyst to surface species and significantly lowering the reaction barrier for C−H bond cleavage from the CH_3_O* and HCOO* intermediates. Ma et al. [57] reported that an optimal Cu^+^/Cu^0^ ratio of approximately 1.00, achieved in a catalyst (Cu/Ce_0.7_Zr_0.3_O_2_, entry 17 in Table 2) with a Ce/Zr ratio of 0.7/0.3, yielded the best performance, with high hydrogen production rates and very low CO selectivity. They found that CH_3_OH molecules were primarily adsorbed/activated on Cu^+^ sites, while H_2_O molecules were predominantly adsorbed/activated at Cu^0^ sites.

Mao et al. [43] performed in situ diffuse reflectance infrared Fourier-transform spectroscopy (DRIFTS) analysis for 10 wt% Cu/Al_2_O_3_ (entry 3 in Table 2) and detected an intermediate formate species adsorbed at the interface at 1602 cm^−1^. This formate species (HCOO–CuAl) was observed to dissociate more rapidly into CO_2_ and H_2_ than the species adsorbed on Al_2_O_3_ (HCOO–Al). Furthermore, the reverse Al_2_O_3_/Cu catalyst provided additional confirmation that the Cu–Al_2_O_3_ interface played a crucial role in MSR. Xu et al. [55] achieved a high H_2_ productivity of 52.8 μmol·g_cat._^−1^·s^−1^ at 200 °C with undetectable CO production, and no deactivation was observed during a 200-hour test when supplying CH_3_OH/H_2_O (1/1, mol/mol) by using an inverse ZrO_2_-0.1/Cu (Zr/Cu molar ratio of 0.1) (entry 15 in Table 2) obtained via oxalate sol-gel co-precipitation followed by calcination/hydrogen reduction treatment. This was attributed to the formation of a specific ZrO(OH)-(Cu^+^/Cu) interfacial structure during the reaction. This highly reactive interfacial -OH group converted HCHO* (produced from methanol at the Cu^+^/Cu site during decomposition, yielding CO/H_2_), via the HCOOH* intermediate, into H_2_ and CO_2_.

Li et al. [47] observed that the migration of ZnO*_x_* species onto the surface of metallic Cu^0^ nanoparticles was facilitated when the commercial Cu/ZnO/Al_2_O_3_ catalyst was exposed to a H_2_/H_2_O/CH_3_OH/N_2_ mixture at 300 °C (entry 7 in Table 2). These more abundant Cu–ZnO*_x_* interfacial sites improved the long-term stability by threefold and enhanced the catalytic activity by twofold for MSR [47]. Li et al. [61] reported that Ga introduction promoted the migration of ZnO*_x_* species to the surface of metallic Cu^0^ nanoparticles after hydrogen reduction at 300 °C and atmospheric pressure, thereby enriching the Cu–ZnO_x_ interface sites (entry 21 in Table 2). This resulted in a 4.6-fold increase in intrinsic activity relative to Cu/ZnO, with negligible strong metal-support interaction (SMSI). Yan et al. [67] observed that adding an appropriate amount of Fe to Cu/ZnO/Al_2_O_3_ promoted the dispersion of Cu and Zn, forming abundant Cu–ZnO_x_ interfacial sites, which enhanced both apparent and intrinsic activity. The best catalyst performance was observed for the CuZnAl-0.1Fe catalyst, achieving a maximum hydrogen production rate of 37.4 μmol·g_cat._^−1^·s^−1^ at 200 °C (entry 27 in Table 2).

Shokrani et al. [48] investigated the effect of alumina content on the properties of a series of CuO/ZnO/Al_2_O_3_ catalysts synthesized by combustion using urea fuel. They reported that increasing the alumina content reduced the crystallinity of Cu and Zn oxides and that adding alumina to the CuO/ZnO catalyst increased the methanol conversion rate while decreasing CO production. They claimed that a catalyst with a molar ratio of CuO/ZnO/Al_2_O_3_ of 4/4/2.5 exhibited the best performance, at 240 °C, without producing CO (entry 8 in Table 2). Sanches et al. [56] reported that in Cu/ZnO/ZrO_2_, the role of ZrO_2_ nanoclusters or amorphous material prevented CuO and ZnO grain growth by inducing microdeformation within the Cu and Zn oxide lattices, thereby promoting the formation of exposed CuO species. These exposed CuO species are readily reducible, enhancing the catalytic performance of Zr-based catalysts (entry 16 in Table 2). They also reported that the monoclinic ZrO_2_ found in Zr-based catalysts exhibited higher CO adsorption capacity, significantly reducing CO production.

Yang et al. [53] investigated the effects of ceria morphology (e.g., nanorods (R), nanoparticles (P), sponge-like structure (S)) and reported that the CuO/CeO_2_-R catalyst exhibited superior catalytic activity compared to CuO/CeO_2_-P and CuO/CeO_2_-S catalysts due to strong interactions between copper oxide and the ceria support (entry 13 in Table 2). This was attributed to high Cu dispersion, low CuO reduction temperature, and high active species Cu^+^ content. Furthermore, it was argued that the oxygen vacancy content on the catalyst surface had a positive effect on reactivity. Cheng et al. [68] reported that the Cu/CeO_2_(CSC) catalyst prepared by the colloidal solution combustion (CSC) method possessed highly dispersed copper species and abundant Cu^+^-O_v_-Ce^3+^ sites at the copper–ceria interface, contributing to an outstanding hydrogen production rate (entry 29 in Table 2). They also explained that the linear correlation between the turnover frequency (TOF) value and the amount of Cu^+^-O_v_-Ce^3+^ sites indicated the crucial role of these sites in the MSR reaction, demonstrating their efficient ability to activate water. The Cu/ZnGa_2_O_4_ catalyst in which 3–4 nm copper particles stabilized on a defective ZnGa_2_O_4_ spinel oxide surface provided a hydrogen productivity of 4.88 μmol·g_cat._^−1^·s^−1^ at 150 °C (entry 20 in Table 2) [60].

Jiang et al. [69] observed the promoting effect of CrO*_x_* and reported that the optimal Cu-7%CrO*_x_*/Al_2_O_3_ catalyst achieved a hydrogen production rate of 317 μmol·g_cat._^−1^·s^−1^ at 260 °C (entry 30 in Table 2). They attributed the enhanced hydrogen production rate and CO_2_ selectivity of Cu-7%CrO*_x_*/Al_2_O_3_ compared to Cu/Al_2_O_3_ to improved Cu dispersion, a Cu^+^/Cu^0^ ratio reaching approximately 1.0, and increased active oxygen species. These factors promoted the rate-determining step (RDS) involving the reaction from CH_3_O* to HCOO* and CO oxidation. He et al. [71] investigated the MSR activity over Cu catalysts supported on ZnZrO_x_ supports with various Zn/Zr ratios and reported that the highest activity was observed over the catalyst with the highest (Cu^0^+Cu^+^) content, the largest Cu active surface area, and the most abundant chemisorbed oxygen and basic sites (entry 32 in Table 2).

**Figure 2:**
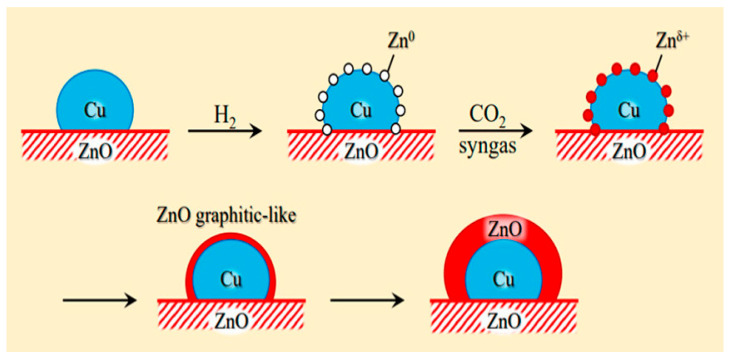
Schematic diagram for different models of Cu-ZnO catalysts under methanol synthesis conditions. The MSR is the reversed methanol synthesis reaction. This schematic diagram is reprinted with permission from ref. [80]. Copyright 2024, American Chemical Society.

Cu/ZnO/Al_2_O_3_ is the most well-known commercial catalyst for MSR. It is also active for methanol synthesis [73,74,75,76,77,78,79,80,81,82] and WGS reaction [83,84]. In addition to its economic advantages over other precious metal-based catalysts, it has high activity and selectivity for CO_2_, especially at low temperatures (200–300 °C). The Cu/ZnO/Al_2_O_3_ catalyst, wherein Cu nanoparticles are dispersed on an alumina support with ZnO particles acting as a promoter, has been employed globally for methanol production for half a century. However, as a dynamic catalyst whose surface structure alters under varying conditions, such as temperature, pressure, and reagent composition, it remains challenging to clearly identify its active sites, necessitating ongoing research. For instance, during methanol synthesis, the formation of CuZn alloys, the migration of Cu nanoparticles onto the ZnO surface (covered by an ultrathin oxide layer), and the role of the Cu–ZnO interface (particularly in the presence of Cu^2+^ ions) have been reported (Figure 2) [80]. Furthermore, it has been proposed that the alumina provides acidic sites that stabilize intermediates or that Al dopants penetrate the ZnO phase, altering its properties [80].

**Figure 3:**
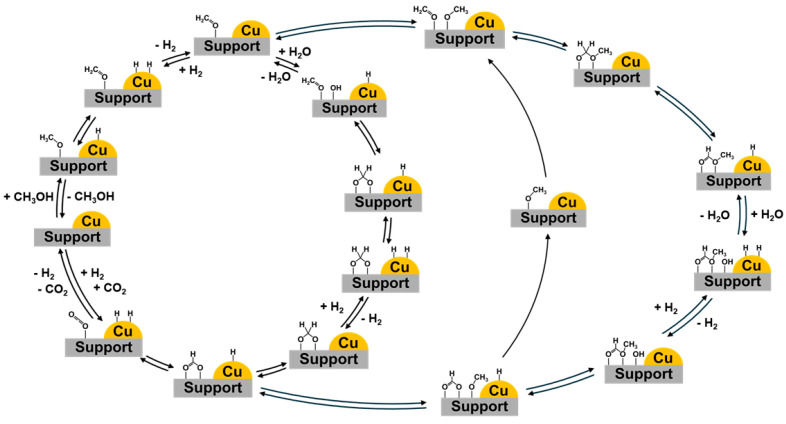
Schematic diagram for the reaction mechanism for MSR over Cu-based catalysts as proposed by Frank et al. [86].

Regarding the reaction mechanism over Cu-based catalysts [85], as shown in Figure 3, Frank et al. [86] proposed comprehensive catalytic cycles, including the HCOO* route (left cycle in Figure 3) and the HCOOCH_3_* route (right cycle in Figure 3). The HCOOCH_3_* route is known to be prevalent in low steam-to-methanol ratios. Recently, a density functional theory (DFT) calculation was conducted to understand the reaction mechanism over different model systems. Meng et al. [42] compared these two routes over Cu(111)/CuAlO_2_(101) structures and reported that the HCOOCH_3_* route was more favorable than the HCOO* route. Li et al. [47] compared the Gibbs free energy diagrams for all elementary reactions involved in the MSR according to the HCOO* route over Cu(111) and Zn_3_O_2_H_2_/Cu(111) and found that both the dehydrogenation of *CH_3_O to *CH_2_O and H_2_O dissociation were facilitated on the Cu–ZnO*_x_* site. Li et al. [87] proposed the reaction pathway for MSR over Cu/ZnO, wherein methanol is adsorbed and dissociated into surface methoxy species, subsequently reacting with hydroxyl generated during water dissociation to form surface formate species, which ultimately decompose to yield CO_2_ and H_2_. Furthermore, they focused on the interfacial sites involved in the methoxy dehydrogenation and water dissociation.

Research on the sintering inhibition of active Cu metal [37,49,88,89,90,91] and regeneration of the spent catalyst [92] is still actively underway. Despite their high activity and economic advantages in MSR, copper-based catalysts still face limitations due to thermal stability issues and restricted applicability under hydrothermal or low-temperature conditions, necessitating ongoing research to overcome these challenges. For this purpose, noble metal-based catalysts have also been used for MSR. Table 3 summarizes some noble metal-based catalysts active in MSR [93,94,95,96,97,98,99,100,101,102,103,104,105,106,107,108,109,110,111,112,113,114,115,116,117,118,119,120,121,122].

Pd-based catalysts (e.g., Pd/ZnO) have been studied for MSR at high temperatures [95,123]. The formation of Pd-Zn alloy has been reported to be responsible for high catalytic activity over Pd/ZnO [96,99]. Li et al. [96] observed that the adsorption energy difference between methanol and water disappeared on ZnPd/ZnO, suggesting that this explained why ZnPd/ZnO exhibited the highest MSR activity (entry 4 in Table 3), as methanol could competitively adsorb and react with water. They also reported that introducing Pd into ZnO reduced the thermodynamic stability of adsorbed formaldehyde (an intermediate in the MSR reaction). However, the resulting ZnPd intermetallic compound strengthens the bond of the adsorbed formaldehyde, enabling further reaction with water. This ultimately drives the reaction pathway toward CO_2_ and H_2_. Liu et al. [98] reported that the octahedral ZnAl_2_O_4_ spinel support, possessing only polar surfaces, provided strong interactions between Pd and Zn, enabling the formation of PdZn_β_ alloy even at low Pd loadings. They also reported that even at 1000 ppm Pd, the PdZn_β_ alloy exhibited catalytic properties similar to those of Cu (entry 6 in Table 3). Zhang et al. [102] attempted to address the low CO_2_ selectivity issue in conventional Pd/ZnO catalysts, where the pathways for the key intermediate CH_2_O* to oxidize into CO_2_ and to decompose directly into CO and H_2_ compete with each other. They succeeded by introducing Cu to lower the water dissociation energy barrier, thereby providing a more active hydroxyl group for CH_2_O* oxidation, while simultaneously raising the CO desorption energy barrier in the PdCu alloy to suppress CH_2_O* decomposition (entry 10 in Table 3).

Gu et al. [104] reported an exceptionally high TOF over single-atom Pt supported on ZnO nanowire (entry 12 in Table 3). Through DFT calculations, they revealed that the catalysis by the single platinum atom coordinated to the lattice oxygen enhanced reaction rates by forming stronger bonds with intermediates, lowering reaction barriers, and altering reaction pathways. Cai et al. [107] reported that adding a small amount of Zn to Pt/MoC not only promoted the formation of the α-MoC_1−x_ phase but also enhanced Pt dispersion and the interaction between α-MoC_1−x_ and Pt active sites, thereby increasing catalytic activity for MSR even at low temperatures (120–200 °C) (entry 15 in Table 3). Gao et al. [110] achieved high TOFs for MSR over Pt/γ-Mo_2_N catalysts (entries 18, 19 and 20 in Table 3), wherein an inert oxide nano-overlay, atomically dispersed on the highly active γ-Mo_2_N surface, blocked the excess surface active sites of γ-Mo_2_N that induce surface oxidation. Wang et al. [124] demonstrated that MSR proceeds via methanol dehydrogenation followed by the WGS reaction over Pt/NiAl_2_O_4_, and argued that the interfacial region and the vacancies on the support (NiAl_2_O_4_) were considered the actual active sites for the methanol dehydrogenation and WGS reaction, respectively.

Au-based catalysts (e.g., Au/CeO_2_ [114,115,116,117,118,119,120,121,122]) are particularly active at low temperatures, making them very feasible for reducing CO concentrations via thermodynamically favorable WGS at low temperatures. However, the high price of gold and uncertainty about the stability of the catalyst are hurdles that need to be overcome for practical application.

Recently, an efficient electrochemical-assisted MSR reaction for pure H_2_ production at lower temperatures (~140  °C) was reported by coupling the electrocatalysis reaction into the MSR in a polymer electrolyte membrane electrolysis reactor [125]. Through electrochemical assistance, the two critical steps, including the methanol dehydrogenation and water-gas shift reaction, are accelerated, which is attributed to decreasing the methanol dehydrogenation energy and promoting the dissociation of H_2_O to OH* by the applied potential [125].

## 3. Aqueous-Phase Reforming of Methanol (APRM)

Various catalysts, including Pt-based, Cu-based and Ni-based catalysts, have been reported to be active for APRM [126,127,128,129]. Table 4 summarizes some noble metal-based catalysts active for APRM [130,131,132,133,134,135,136,137,138,139,140,141,142,143,144,145,146,147,148,149,150,151,152,153,154,155,156,157,158,159].

Lin et al. [130] achieved a high TOF over Pt atomically dispersed on α-molybdenum carbide (α-MoC) (entries 1 and 2 in Table 4). This was attributed to the exceptional water-splitting induction capability of α-MoC and the synergistic effect between Pt and α-MoC in activating methanol. Li et al. [132] compared the performance of Pt/NiAl_2_O_4_ spinel (entry 5 in Table 4) and Pt/γ-Al_2_O_3_ (entry 6 in Table 4), observing that the former exhibited significantly superior APRM activity and stability compared to the latter. For the Pt/NiAl_2_O_4_ catalyst, the presence of oxygen vacancies facilitated the reduction of PtO_x_ to metallic Pt, leading to higher catalytic performance in the methanol dehydrogenation reaction. Furthermore, the redox mechanism dominated the WGS reaction on the Pt/NiAl_2_O_4_ catalyst, providing a faster WGS reaction rate compared to the coupling pathway observed on Pt/γ-Al_2_O_3_. Leng et al. [133] demonstrated that nitrogen doping significantly enhanced the catalytic performance of 0.5 wt% Pt/CeO_2_ (entry 7 in Table 4), increasing the TOF from 773 to 1290 h^−1^ at 200 °C compared to the undoped sample. This was interpreted as resulting from increased oxygen vacancies due to the formation of Ce-N-O bonds. Gong et al. [136] achieved a high TOF of 14,813 h^−1^ over a 0.2 wt% Pt/γ-Mo_2_N(O_0.3_) catalyst (entry 10 in Table 4) at 210 °C, where atomically dispersed Pt species were stably anchored on the unique γ-Mo_2_N(O_0.3_) surface. They suggested that Pt species dominated methanol activation and reforming processes, while the interface between Pt and the γ-Mo_2_N framework modified with partial MoO_x_ species (i.e., γ-Mo_2_N(O_0.3_)) played a pivotal role in accelerating water dissociation kinetics.

Li et al. [137] combined the photocatalytic and thermocatalytic processes and obtained a H_2_ production rate (5.66 μmol·g_cat._^−1^·s^−1^) over a 0.05%Pt@TiO_2_ catalyst, which was far higher than those for the thermocatalytic process (1.89 μmol·g_cat._^−1^·s^−1^, entry 11 in Table 4) and the photocatalytic process (0.80 μmol·g_cat._^−1^·s^−1^), respectively. This was attributed to the fact that the photo-generated holes and hydroxyl radicals enhanced methanol dehydrogenation, water molecule splitting, and the water–gas shift reaction, while high temperature accelerated reaction kinetics. Huang et al. [138] compared three Pt catalysts supported on rod-shaped ceria (CeO_2_-R), cubic ceria (CeO_2_-C), and octahedral ceria (CeO_2_-O), observing that Pt/CeO_2_-R exhibited the highest hydrogen production rate (entry 12 in Table 4), which was explained by the highly dispersed Pt on the CeO_2_-R, providing a substantial number of active metal sites and promoting the efficient adsorption and activation of methanol. They also discovered a strong correlation between the TOF and the presence of oxygen vacancies on Pt/CeO_2_, indicating that abundant oxygen vacancies on the catalyst enhance substrate adsorption and promote the rapid conversion of CO* adsorbed on platinum, thereby accelerating the WGS reaction via faster redox pathways. This led to higher hydrogen production at lower CO selectivity. Tian et al. [151] reported that the Pt/Ce_0·5_Mg_0·5_O_2_ catalyst enhanced CO* oxidation and APRM activity by leveraging the advantages of both oxygen vacancies that promoted H_2_O adsorption/decomposition and strong basic sites that contributed to formate group (HCOO^−^) formation (entry 27 in Table 4).

Li et al. [149] synthesized Pt/Fe_5_C_2_@C, composed of Fe_5_C_2_ nanoparticles encapsulated in graphite carbon layers (Fe_5_C_2_@C) along with loaded Pt, and obtained a high H_2_ production rate of 38.9 μmol·g_cat._^−1^·s^−1^ at 200 °C over 11%Pt/Fe_5_C_2_@C (entry 25 in Table 4) owing to SMSI between Pt and Fe_5_C_2_@C. Zhang et al. [154] demonstrated that the dual-active sites of Pt single atoms and frustrated Lewis pairs (FLPs) on porous nanorods of CeO_2_ enabled H_2_ generation with low CO (0.027%) at 120 °C (entry 30 in Table 4). They explained that this high activity stemmed from the spontaneous water dissociation on FLPs, which enhanced the reforming reaction of *CO by platinum single atoms, thereby promoting H_2_ production and suppressing CO formation [154]. Wang et al. [155] proposed cooperative triactive sites of atomically dispersed Pt, adjacent oxygen vacancies (O_V_) on In_2_O_3_, and hydroxyl-saturated six-coordinated In_6_ on Pt_1_/In_2_O_3_ (entry 31 in Table 4) and achieved near-CO-free H_2_ generation (<1 ppm) through APRM at low temperatures (<180 °C). They claimed that the incorporation of In_6_ sites with high dehydrogenation capability ensured rapid *H depletion, effectively suppressing CO release [155]. Arooj et al. [148] observed a high TOF of 96.9 h^−1^ at 100 °C over a highly defective catalyst (Pt/H_2_-In_2_O_3_) (entry 24 in Table 4), which was attributed to the synergistic effect of the uniformly dispersed Pt nanoparticles with higher electron density and defect-rich In_2_O_3_ support, promoting the adsorption and activation of reactant molecules and accelerating the reaction kinetics.

Pt-based catalysts, including Pt/Al_2_O_3_ [130,131,132,134,135,139], Pt/C [146], Pt/ZrO_2_ [143], and Pt/CeO_2_ [133,138], are highly active and selective for hydrogen production as well as resistant to coking and sintering at low temperatures. Among them, Pt catalysts supported on ceria-containing supports with oxygen storage capacity enhance water activation [133,138]. However, their limited availability and high price are inevitable drawbacks [126].

Comparatively, other Group VIII metal-based catalysts, including Pd, Rh, Ru, and Ni, were less active towards conversion of oxygenated compounds or more selective towards alkane formation due to their high C−O bond cleavage activity [127,129,156]. Furthermore, the acidity of the support also strongly influenced H_2_ selectivity via dehydration reactions involving C−O bond cleavage [156,159].

To overcome the limitations of the monometallic catalysts, bimetallic catalysts (e.g., Pt–Ni, Pt–Co, and Pt–Re) were proposed to improve the catalytic performance and enhance the resistance to deactivation. Pt–Ni/C shows higher H_2_ yield than Pt alone [126]. Pt-Ru-based catalysts were reported to exhibit high activity for the APRM. Among Pt-Ru catalysts using various supports (SiO_2_, TiO_2_, Al_2_O_3_, MgO, CeO_2_, and ZrO_2_), the Pt-Ru/TiO_2_ catalyst exhibited the most favorable performance in terms of CO_2_ selectivity and activity [156,157,158,159]. This was interpreted as being due to the SMSI phenomenon and Pt-Ru alloy formation, respectively. Tian et al. [140] observed a continuous decrease in CH_4_ selectivity, with increasing Fe content on PtFe/Al_2_O_3_ catalysts (entry 14 in Table 4). This was interpreted as the indirect WGS reaction via lattice oxygen in FeO_x_ species promoting WGS reactivity, thereby enhancing H_2_ selectivity in the APRM. Na et al. [143] demonstrated that introducing Na into the ZrO_2_ lattice formed an electron-rich ZrO_2_ surface, promoting the creation of low-coordination Pt sites adjacent to Na species. These sites favored CO adsorption for the WGS reaction, enabling Pt/Na–ZrO_2_ (entry 18 in Table 4) to exhibit superior performance compared to Pt/ZrO_2_. Wang et al. [153] reported that K^+^-doped Pt nanoparticles (PtK_x_/Al_2_O_3_) on the γ-Al_2_O_3_ phase stabilized the *OH intermediate on the Pt surface, achieving a TOF of 142.3 h^−1^ at undetectable CO concentrations (below 5 ppm) at 120 °C (entry 29 in Table 4). They explained this was because K^+^ shifted the d-band center within the Pt nanoparticles, stabilizing the *OH generated from water dissociation without interfering with methanol dissociation. Jia et al. [147] reported that the hexagonal close-packed platinum-tin intermetallic compound (PtSn/C_3_N_4_) exhibited high APRM activity, with a TOF of 56,024 h^−1^ at 200 °C (entry 23 in Table 4). Furthermore, they observed that the H_2_ productivity of PtSn/C_3_N_4_ was 1.5 times that of Pt_3_Sn/C_3_N_4_. This was attributed to PtSn/C_3_N_4_ having a shorter Pt–Sn bond length and a lower Pt–Pt coordination number compared to Pt_3_Sn. Consequently, it suppressed the formation of *CO and H_2_O* (*CO···H–OH*) formation, weakening the co-adsorption of *CO and H_2_O* and reducing the additional formation energy requirement for HCOO* (0.60 vs. 0.92 eV), which was the RDS in the APRM.

Among cost-effective and widely available transition metal catalysts, Ni- and Cu-based catalysts have mainly been reported. Table 5 summarizes some transition metal-based catalysts active for APRM [131,160,161,162,163,164,165,166,167,168,169,170,171,172,173,174,175,176,177,178,179,180,181,182,183].

Ni-based catalysts, including Ni/Al_2_O_3_ and Ni/CeO_2_, have good activity, especially with promoters or supports (e.g., CeO_2_ and ZrO_2_). However, they are prone to sintering and coke formation and have lower hydrogen selectivity compared to Pt catalysts [160,162]. Xiao et al. [164] synthesized a series of Ni catalysts supported on basic mixed metal oxides (Ni_x_Mg_y_-mixed metal oxides) from a NiMgAl layered double hydroxide (LDH) precursor, yielding high APRM activity (entry 6 in Table 5). For this catalyst, the intrinsic activity for methanol decomposition increased significantly with decreasing Ni particle size, while the WGS and APRM reactions were primarily enhanced by the intermediate basicity derived from Mg-O pairs [164].

**Figure 4:**
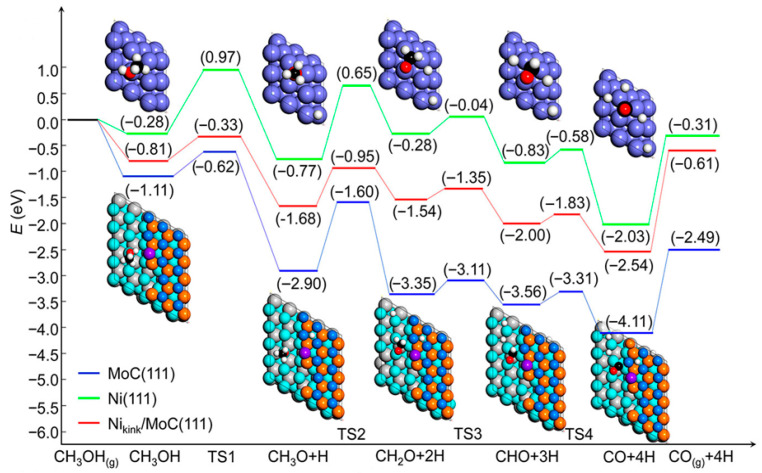
Energy profiles for CH_3_OH dissociation into CO and H atoms on α-MoC(111), Ni(111), and Ni_kink_/α-MoC(111) surfaces. This is reprinted with permission from ref. [131]. Copyright 2020, American Chemical Society.

Lin et al. [131] reported that the H_2_ production rate of 2% Ni/α-MoC (entry 7 in Table 5), in which Ni was atomically dispersed over α-MoC via carbon bridge bonds, forming a Ni_1_–C*_x_* motif on the carbide surface, was about six times higher than that of the 2% Pt/Al_2_O_3_ catalyst. According to their detailed reaction pathway calculations (Figure 4), the RDS on the models is the second step, *CH_3_O → *CH_2_O + *H for methanol decomposition (Figure 4). The energy barrier on Ni_kink_/α-MoC(111) (0.73 eV) is significantly lower than that of α-MoC(111) (1.30 eV) and Ni(111) (1.42 eV). This indicates that while pure α-MoC and Ni catalysts are unfavorable for methanol activation, Ni/α-MoC with a Ni_1_–C*_x_* motif is efficient at this stage. Additional DFT calculations on isolated nickel atoms or nickel metal clusters of varying sizes at different sites on α-MoC(111) could provide insights for designing more active catalysts. Xiao et al. [166] reported that during APRM using the Ni@NC catalyst, adding KOH at 240 °C increased the hydrogen production rate from 70.7 (entry 9 in Table 5) to 406 μmol H_2_·g_cat._^−1^·s^−1^ (entry 10 in Table 5), while CO selectivity decreased from 16.5 to 0.2%. They reported that KOH primarily reacted directly with CO generated during methanol dehydrogenation to form HCOOK, thereby eliminating the toxic effect of CO on active nickel sites and significantly promoting methanol dehydrogenation [166]. Additionally, a small amount of KOH was also reacted with CO_2_ to promote WGS [166].

Cu/ZnO/Al_2_O_3_, commonly used in methanol steam reforming, was also adapted for APRM. However, it has lower stability in aqueous environments and is sensitive to oxidation and leaching [180]. Therefore, it needs structural promoters for improved performance in APRM. Li et al. [169] prepared the Cu/ZnO/CeO_2_ catalyst with high-density ultra-small Cu nanoparticles and abundant oxygen vacancies and reported that the optimized 55% Cu/ZnO/CeO_2_ catalyst (Cu nanoparticles of 3.8 nm, Cu loading of 55.89 wt%, oxygen vacancies of 2.316 × 10^15^ spins·g_cat._^−1^) exhibited an excellent H_2_ evolution rate of 58.39 μmol·g_cat._^−1^·s^−1^ even at low temperature of 210 °C (entry 13 in Table 5), which was a 2.1-fold enhancement over that of the commercial Cu/ZnO/Al_2_O_3_ catalyst.

Lu et al. [170] prepared Ga_2_O_3_-modified Cu–ZnO catalysts with carbon encapsulation and demonstrated that the best catalyst, 12 wt % GCZ-2.0-C450 (entry 14 in Table 5), exhibited a high H_2_ production rate of 101.2 μmol·g_cat._^−1^·s^−1^ and low CO selectivity (0.07%) at 210 °C, with long-term stability. They explained that the introduction of Ga_2_O_3_ significantly reduced the Gibbs energy of the rate-determining step in the reaction *CH_3_O + *H → *CH_2_O + *H_2_ from 1.25 eV to 0.60 eV, and that the adsorption energy of the generated *H_2_ decreased from −2.92 eV to −0.13 eV, thereby promoting the release of gaseous H_2_ [170]. Liu et al. [172] synthesized Cu/CuO_x_/C catalysts by encapsulating Cu/CuO_x_ nanoparticles within a carbon matrix, utilizing Cu-benzene-1,3,5-tricarbocylate (BTC) as a precursor, and obtained a hydrogen evolution rate of 33.7 μmol·g_cat._^−1^·s^−1^ at 210 °C (entry 16 in Table 5). Lu et al. [178] synthesized a Cu-based catalyst using Cu, Cu_2_O, and CuN_3_ nanoparticles immobilized on nitrogen-doped carbon (entry 22 in Table 5), forming Cu^0^/Cu^+^/Cu-N_3_ active sites and achieving excellent catalytic performance (e.g., high H_2_ production rate and outstanding long-term stability). They explained that the Cu-N_3_ active site promoted the decomposition of *OH ions in water and enhanced the conversion rates of *CO and *OH, thereby improving CO conversion and hydrogen production rates. The Cu^0^/Cu^+^ catalyst encapsulated in the high content of graphited N-doped carbon (e.g., Cu@NGC-600, entry 15 in Table 5) gave an excellent hydrogen production rate of 46.15 μmol·g_cat._^−1^·s^−1^ at 190 °C, which was four times higher than the commercial 20% Pt/C catalyst [171]. Lu et al. [180] employed a carbon layer-encapsulated hierarchical porous microsphere strategy to synthesize the Cu-SP/Al_2_O_3_–ZnO (Cu-SP/AZ) catalyst (entry 24 in Table 5). This enhanced copper dispersion, enabling a high hydrogen production rate of 64.2 μmol·g_cat._^−1^·s^−1^ at 210 °C, which was nearly equivalent to that of the 20 wt% Pt/C catalyst.

Fe–Cu nanoparticles embedded within the nitrogen-doped graphitic carbon matrix (entry 27 in Table 5) were also reported to be active (TOF= 317 h^–1^) and selective (no CO detected) catalysts for APRM with stoichiometric H_2_O amounts [183].

## 4. Aqueous Methanol Dehydrogenation (AMDH)

AMDH is composed of a series of dehydrogenation reactions, as follows.


CH_3_OH(l) → HCHO(l) + H_2_(g) (8)

HCHO(l) + H_2_O(l) → CH_2_(OH)_2_(l) (9)

CH_2_(OH)_2_(l) → HCOOH(l) + H_2_(g) (10)

HCOOH(l) → CO_2_(g) + H_2_(g) (11)

Various homogeneous catalyst-based systems have been reported [24,184,185,186,187,188,189]. Table 6 summarizes some homogeneous catalysts reported for AMDH [190,191,192,193,194,195,196,197,198,199,200,201,202,203,204,205,206,207,208,209,210,211,212,213,214,215].

The Beller group [195] reported an efficient AMDH process at low temperatures catalyzed by a ruthenium complex exhibiting an excellent catalytic TOF (4700 h^−1^) and turnover number (over 350,000) at 65–95 °C under atmospheric pressure conditions (entries 6, 7, and 8 in Table 6). Milstein and co-workers [196] demonstrated that a Ru-PNN pincer complex (entry 9 in Table 6) was also active for this reaction in the presence of two equivalents of NaOH in MeOH/H_2_O/toluene (0.4/1/0.5 mL) solution at mild temperature (<100 °C); this catalyst solution could be reused without separation or purification, and no decrease in catalyst activity was observed for approximately one month [196]. The Beller group reported a bi-catalytic system (entry 11 in Table 6) of Ru-MACHO-BH, [RuH(BH_4_)(CO)(HN(C_2_H_4_PPh_2_)_2_)], and [Ru(H)_2_(dppe)_2_] work in a synergistic manner to generate hydrogen from aqueous methanol even in the absence of a base [198].

**Figure 5:**
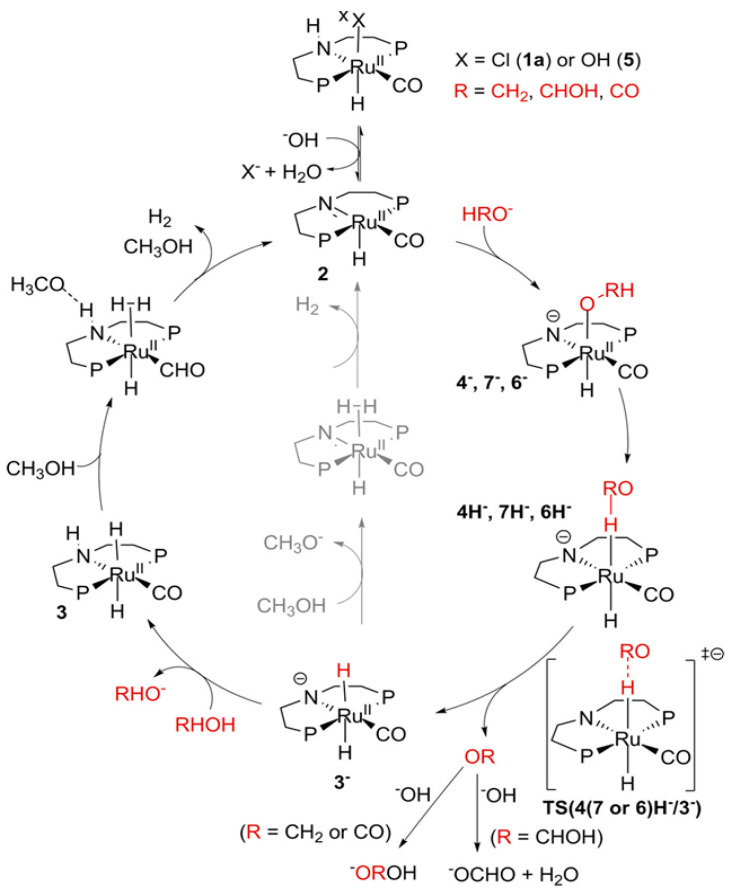
Proposed catalytic cycle for low-temperature methanol reforming catalyzed by ruthenium PNP complex (**1a**), (RuH(CO)Cl(HN(C_2_H_4_P*i*-Pr_2_)_2_)) (P = *i*-Pr_2_). ^‡^ denotes transition state. This is reprinted with permission from ref. [216]. Copyright 2016, American Chemical Society.

Beller and co-workers [216] described the reaction mechanism (Figure 5) for AMDH in the presence of a ruthenium PNP complex, (RuH(CO)Cl(HN(C_2_H_4_P*i*-Pr_2_)_2_)) (entry 7 in Table 6). According to their report, Ru–amido (**2**) exhibits high reactivity with methanol, formic acid, and water, forming ruthenium mono and dihydride complexes in an equilibrium state that can be disrupted by a base (dihydride (**3**)) or an acid (monohydride (**4**)). During catalysis, the O- and CH- coordination modes of the methoxide toward ruthenium compete, forming Ru–monohydride (**4^-^**) and Ru–dihydride (**3^-^**) complexes, respectively. Increasing KOH not only accelerates the reaction rate but also increases the **3^-^**/**4^-^** ratio, demonstrating that the “inner-sphere” C–H cleavage via C–H coordination of the methoxide to ruthenium is promoted by the base. Protonation of **3^-^** releases H_2_ gas and formaldehyde, with the latter being rapidly consumed by KOH to generate the corresponding gem-diolate, which provides the overall driving force for the reaction. The complete methanol reforming reaction is achieved through this corresponding step initiated from the gem-diolate and formate.

The Grützmacher group independently reported methanol dehydrogenation at neutral pH by a homogeneous host-guest type ruthenium complex (entry 10 in Table 6) [197]. Qi et al. [204] synthesized two series of Ru(II) complexes (entry 17 in Table 6) with lutidine- and pyridine-linked bis-*N*-heterocyclic carbene pincer ligands and found that ([Ru(L_mes_)Cl_2_(CO)], where L_mes_ is the bis-NHC ligand with a mesityl functional group) produced a TOF of 89 h^–1^ at 94 °C. Reek and co-workers prepared a Ruthenium-carbonyl complex with a salen-type ligand (entry 15 in Table 6) and claimed that the carbonyl complex was not the inactive species; rather, the carbonyl ligand (derived from methanol) undergo attack by base/water to form formate [202].

Zhou and co-workers [208] demonstrated that a homogeneous catalyst [Cp*Rh(NH_3_)(H_2_O)_2_]^3+^ (entry 21 in Table 6) could produce hydrogen via AMDH under ambient pressure at 70 °C; the corresponding TOF is 83.2 h^−1^ without any additional alkaline or organic substances. Fujita et al. [210] reported an anionic iridium complex (entry 23 in Table 6) bearing a functional bipyridonate ligand as a catalyst for methanol dehydrogenation under mild conditions (weakly basic solution below 100  °C) without the use of an additional organic solvent. Zhou utilized 6-hydroxypicolinic acid ligand for the Ir(III) complex (entry 25 in Table 6), which showed a TOF of 377 h^−1^ at 78 °C using a 1.58 mM concentration of weak base Na_2_CO_3_ [212].

The Beller group first reported on non-noble metal-catalyzed AMDH [213]. They achieved a TOF of 702 h^−1^ at 91 °C under strongly basic conditions using iron metal with a PNP pincer ligand, [FeH(BH_4_)(CO)(HN(C_2_H_4_PiPr_2_)_2_)] (entry 26 in Table 6), which was stable and generated hydrogen for 5 days in the presence of an excess amount of ligand [213]. Holthausen and co-workers reported that the iron-pincer complex (entry 27 in Table 6) with co-catalyst LiBF_4_ (Lewis acid) was able to dehydrogenate methanol completely [215]. They described that the presence of Lewis acid helped in the generation of catalytically active species by promoting the Fe-formate complex’s decarboxylation. The Beller group reported the first example of AMDH using an Mn-PNP complex (entry 28 in Table 6), with a stability of more than a month [214].

In homogeneous catalysts for AMDH, multi-coordinate ligands with three or more coordination sites are typically used. This prevents formaldehyde from decomposing into CO and instead converts formaldehyde into formate in the presence of water and a base, generating additional hydrogen molecules. Furthermore, since expensive phosphine ligands are primarily used, the discovery and design of new, cost-effective ligands to replace them is necessary.

Heterogeneous catalysts possess distinct advantages over homogeneous catalysts in terms of practical application, even if some loss occurs in the selectivity of the target product [217]. Lu et al. [218] conducted a comparative analysis of three systems of Pt_1_/CeO_2_(110)), Pt_7_/CeO_2_(110), and Pt_1_/Ce_1−*x*_O_2_(110) for AMDH via DFT calculations and revealed that only Pt_1_/Ce_1−*x*_O_2_(110) was favorable for AMDH at low temperatures (100 °C), concluding that both the small size and the Ce vacancy-substituted sites of Pt contribute to the enhanced performance of the Pt/CeO_2_ catalyst. Guo et al. [219] demonstrated that the Pt nanoparticles supported on porous nanorods of CeO_2_ (PN-CeO_2_) with abundant oxygen vacancies (Pt/PN-CeO_2_) enabled the efficient H_2_ generation at 60 °C, with a TOF value of 173.5 h^−1^, and suppress CO generation in the presence of 8 M KOH. At a temperature of 90 °C over the same catalyst, the hydrogen production rate and TOF increased to 20.4 μmol H_2_·g_cat._^−1^·s^−1^ and 1433 h^−1^, respectively. This was because the oxygen vacancies on PN-CeO_2_ could promote H_2_O activation and also enhance the electronic density of supported Pt nanoparticles through SMSI, thereby promoting the activation of methanol. Liu et al. [220] reported a heterogeneous catalyst composed of an iridium cluster and a single atom, wherein the iridium cluster promotes methanol dehydrogenation to produce formic acid, while the adjacent iridium single atom promotes the rapid decomposition of formic acid, converting it into H_2_ and CO_2_ and thereby suppressing the CO intermediate. This achieved a remarkable hydrogen production rate of 346.9 mol H_2_·mol Ir^−1^·h^−1^ or 16.8 μmol H_2_·g_cat._^−1^·s^−1^ and 100% H_2_ selectivity at 95 °C on the optimal catalyst in the presence of 8 M KOH, whilst performing the reaction without detectable CO production. However, it should be noted that no hydrogen evolution was observed in the absence of KOH.

## 5. Summary

**Figure 6:**
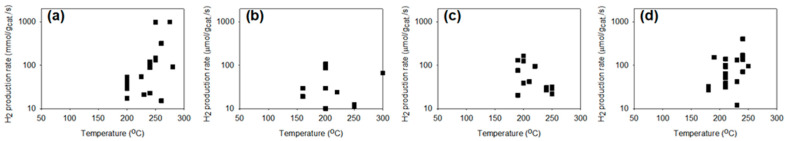
The specific reaction rates for H_2_ production at different temperatures over Cu-based MSR catalysts (**a**), non-Cu-based MSR catalysts (**b**), noble metal APRM catalysts (**c**), and non-noble metal APRM catalysts (**d**).

This review addressed two distinct pathways for hydrogen production from methanol: methanol reforming and methanol dehydrogenation. The methanol reforming reaction can be carried out via heterogeneous catalysts in either the gas phase (MSR) or liquid phase (APRM), whilst methanol dehydrogenation can proceed at the lowest temperatures (e.g., below 100 °C) primarily in the presence of homogeneous organometallic catalysts. The specific reaction rates presented in Table 2, Table 3, Table 4 and Table 5 are compared in Figure 6. In terms of specific reaction rate, the Cu-based catalysts for MSR demonstrate significantly superior performance compared to the other catalysts, such as non-Cu-based MSR catalysts and APRM catalysts. Notably, noble metal catalysts exhibit similar specific reaction rates for both MSR and APRM. For APRM, non-noble metal catalysts exhibit specific reaction rates similar to those of noble metal catalysts despite having a higher active metal content.

**Figure 7:**
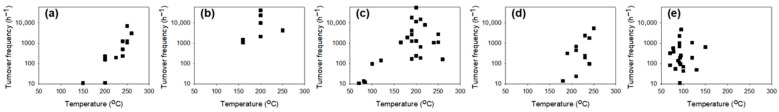
The TOFs for H_2_ production at different temperatures over Cu-based MSR catalysts (**a**), non-Cu-based MSR catalysts (**b**), noble metal APRM catalysts (**c**), non-noble metal APRM catalysts (**d**), and homogeneous AMDH catalysts (**e**).

The intrinsic catalytic activity of various catalysts for hydrogen production from methanol can also be compared based on their TOFs. As shown in Figure 7, the noble metal catalysts exhibit significantly higher TOFs than the Cu-based catalysts. Interestingly, these noble metal catalysts exhibit similar TOFs regardless of whether they are used for MSR or APRM. For APRM, the noble metal-based catalysts demonstrate significantly higher TOFs than non-noble metal catalysts. It is also worth noting that homogeneous catalysts for AMDH exhibit TOFs similar to other catalysts for MSR and APRM, even at much lower temperatures.

Therefore, it can be concluded that Cu-based catalysts are most advantageous for MSR not only in terms of reaction activity but also economic viability. Conversely, noble metal-based catalysts remain the most promising for APRM. For the AMDH catalyst, most reaction data were obtained at temperatures below the boiling point of the reactants under atmospheric pressure. Nevertheless, since these AMDH catalysts exhibit significantly higher TOF compared to Pt-based APRM catalysts under similar reaction conditions, it is considered meaningful to confirm the catalytic activity and stability of homogeneous AMDH catalysts under APRM conditions. Recent studies on heterogenous AMDH catalysts suggest the potential for applying these catalysts under APRM reaction conditions [219,220].

Given the current state of technology, Cu-based catalysts are the most desirable in terms of catalytic activity for hydrogen production from methanol; however, the most economical catalytic process may vary depending on the reaction conditions. For example, in large-scale hydrogen production processes, where energy integration is possible, it is considered most economical to integrate a steam reforming reactor using Cu-based catalysts, a WGS reactor, and hydrogen separation devices such as those using pressure swing adsorption. However, when the methanol content is low, gas-phase steam reforming requires excessive energy for the vaporization of reactants. In particular, for application in distributed systems where hydrogen production volumes are not large, APRM―which does not require an additional hydrogen purification process―becomes more economical than MSR [23]; this is the reason why catalyst research on APRM and AMDH continues.

## 6. Outlook

Based on the catalytic performance of each catalyst for different hydrogen production routes, the future research direction can be proposed as follows. Since the Cu-based catalysts are the most effective for MSR not only in terms of reaction activity but also in terms of economic viability, the future MSR catalyst research should focus on further enhancing the activity and durability of Cu-based catalysts. The efficient utilization of Cu present within the catalyst will be particularly important. For this, the Cu catalyst must contain Cu metal with a highly precisely controlled nanostructure. Specifically, it is desirable from the perspective of the reaction mechanism that Cu metal nanoclusters and Cu^+^ are arranged in close proximity. For Cu^+^, since it can form at the interface between Cu metal and the support, a synthesis strategy is required to provide an abundant interface between them. It will also be necessary to optimize the ratio between Cu metal and Cu cations through appropriate pretreatment. The search for structural promoters that stabilize Cu nano clusters and Cu metal-support interfaces, and electronic promoters that regulate the electronic states of Cu metal and Cu cations at the interface, can be accelerated through theoretical model calculations.

MSR is an endothermic reaction, thus requiring an external energy supply. Traditionally, carbon-based fuels were combusted for this purpose; however, to reduce CO_2_ emissions, direct or indirect electric heating systems utilizing renewable energy sources (e.g., solar, wind, tidal, geothermal energy) can be applied [221,222,223]. The development of suitable catalyst systems for each of these respective systems is necessary. In particular, catalysts specifically designed to selectively heat only the active sites without heating all reactants and the catalyst bed are promising in terms of efficient electrical utilization.

In addition to the thermocatalytic methanol reforming primarily covered in this review, photothermal MSR, combining photochemical effects with photothermal conversion, is also currently being actively researched. It is reported that this enables MSR operation under mild conditions through the synergistic effect of photons and phonons, significantly lowering reaction temperatures, reducing external heat demand, and enhancing reaction rates and selectivity [137,224]. Fu et al. [135] observed that Al_2_O_3_-supported nano-sized Pt photocatalysts enabled efficient hydrogen production (6.912 mmol·g_cat._^−1^·h^−1^) through APRM reaction under light irradiation at low temperatures of 150 °C, which was six times higher than that of APRM reaction conducted in the dark. They demonstrated that the Pt particle size greatly affected the light absorption ability so that the medium Pt particle size (~4.1. nm) enabled lower electron density states, which was beneficial for the dehydrogenation of methanol and the sequential WGS reaction.

Photocatalytic methanol reforming and photocatalytic water splitting using methanol as a sacrificial agent are also being actively researched [225]. In the photocatalytic dissociation of water, electrons and holes generated by light irradiation combine with protons and hydroxide ions in water to produce H_2_ and O_2_, respectively. In the presence of sacrificial reagents such as methanol, excess photogenerated holes are trapped and oxidized, thereby enhancing overall hydrogen productivity. However, in this case, methanol is just completely oxidized into CO_2_ and H_2_O. If the selective oxidation of methanol can be achieved on this side, the simultaneous production of hydrogen and value-added products from a water-methanol mixture becomes possible. Until now, various photocatalysts, including the PtCu–TiO_2_ sandwich photocatalyst [226], MgO nanocrystals [227], single-layer MoS_2_ nanosheets [228], silver nanoparticle (AgNP)/g-C_3_N_4_ aerogel [229], and the Pt–C/TiO_2_ photocatalyst with Pt quantum dots (Pt_QD_) and C-coordinated Pt single atoms (Pt_SA_) [230], have been reported for this purpose. The co-production of hydrogen from water and value-added products from methanol in the presence of visible light will be another direction for photocatalytic methanol utilization [231,232,233].

Regarding APRM catalysts, single-atom catalysts and metal sub-nano clusters represent areas requiring further research. Theoretical calculations based on the APRM reaction mechanism for various combinations of single atoms and their environmental configurations can provide a new set of promising candidate materials. A similar approach could also be applied to the exploration of metal sub-nanoclusters. The next challenge will be finding a way to maximize the surface density of these well-designed active sites without compromising the optimal structure. Catalyst stability under APRM conditions must also be considered during catalyst design.

Regarding AMDH catalysts, as pointed out by Beller’s research team [185], for practical application, the currently known catalytic performance must be enhanced by at least three orders of magnitude. Therefore, seeking combinations of abundant metals and inexpensive ligands necessitates fundamentally new developments at a completely novel level. A DFT calculation can provide insights into the key electronic and structural features of transition metal-based AMDH catalysts for enhancing the catalytic performance [234]. The heterogenized organometallic AMDH catalysts and heterogeneous AMDH catalysts can be considered as potential solutions to address the current limitations of AMDH catalysts. Recent advances in heterogeneous catalysts for AMDH also warrant attention, and computational chemistry plays a significant role in the design of such catalysts. The use of bases, often cited as a problem in the AMDH reaction, can be addressed from a continuous process perspective. Therefore, while benefiting from the significant increase in reaction rate due to base usage, the optimization of catalyst design and operating conditions must not compromise catalyst stability. The fact that the TOF increases as the concentration of the AMDH catalyst decreases suggests that mass transfer limits the overall reaction rate. Reactor design, as well as catalyst development, is deemed necessary for future AMDH process research.

If the goal is to generate electricity using methanol, a direct methanol fuel cell (DMFC) might seem preferable to a system that produces hydrogen from methanol and then generates electricity via a proton exchange membrane fuel cell (PEMFC), as the DMFC does not require an additional reformer. However, the current technological level of DMFCs results in lower overall energy efficiency compared to PEMFCs equipped with methanol reformers, limiting their application to small-scale power generation systems at present [235,236]. Solving the methanol crossover problem and developing more affordable catalysts are critical challenges that must be addressed in DMFC technology [237,238,239,240].

## Figures and Tables

**Table 1:** Comparison of some potential hydrogen production routes.

Entry	Feedstock	Available Hydrogen Content in the Feedstock (wt%)	Representative Reaction to Produce Hydrogen	$∆G_{298K}^{0}$ (kJ)	$∆H_{298K}^{0}$ (kJ)
1	Methane (CH_4_)	25	CH_4_(g) + 2H_2_O(g) ↔ CO_2_(g) + 4H_2_(g)	113	165
2	Methanol (CH_3_OH)	12.6	CH_3_OHg) + H_2_O(g) ↔ CO_2_(g) + 3H_2_(g)	−3.83	49.0
3	Carbon monoxide (CO)	0	CO(g) + H_2_O(g) ↔ CO_2_(g) + H_2_(g)	−28.6	−41.2
4	Formic acid (HCOOH)	4.4	HCOOH(g) ↔ CO_2_(g) + H_2_(g)	−43.2	−14.3
5	Methyl cyclohexane (C_6_H_11_-CH_3_)	6.2	C_6_H_11_-CH_3_(g) ↔ C_6_H_5_-CH_3_ (g) + 3H_2_(g)	150	205
6	Ammonia (NH_3_)	17.8	NH_3_(g) $↔\frac{1}{2}$N_2_(g) + $\frac{3}{2}$H_2_(g)	16.5	46.1

**Table 2:** Some Cu-based catalysts for steam reforming of methanol.

Entry	Catalysts	Preparation Method	Reaction Conditions			Catalyst Performance					Ref.
			Reaction Temperature (°C)	Feed Composition (H_2_O/CH_3_OH)	Space Velocity ^a^	CH_3_OH Conversion (%)	Selectivity to CO (S_CO_) (%)	Production Rate of H_2_ (μmol H_2_·g_cat._^−1^·s^−1^)	TOF ^b^ (h^−1^)	Stability	
1	5 wt% Cu/Al_2_O_3_	Wet impregnation	200	1.5	WHSV_methanol_ = 2.32 h^−1^		1.3 ^c^	17.4	229		[41]
2	4.25Cu/Cu(Al)O_x_ (54 wt% Cu)	Co-precipitation	240	2	GHSV = 21.0 L·g_cat._^−1^·h^−1^	99.5	<2 ^c^	111	234	14% drop in CH_3_OH conversion after 300 h	[42]
3	10 wt% Cu/Al_2_O_3_	Incipient wetness impregnation	250	1	WHSV = 10.56 h^−1^	89.7	0.9 ^d^	148	1080	10% drop in H_2_ production rate after 100 h at 200 °C	[43]
4	5% Cu_5_Zn_10_Al (5 wt% CuO, Zn/Al = 5/10)	Wet impregnation	350	2	GHSV = 15,500 h^−1^	98	0	16.7			[44]
5	CuZn/γ-Al_2_O_3_/Al (9.71 wt% Cu, 1.43 wt% Zn)	Molten salt impregnation	275	2	GHSV = 4.00 L·g_cat._^−1^·h^−1^	100	3.34 ^d^	994		10% drop in CH_3_OH conversion after 100 h	[45]
6	Cu/ZnO/Al_2_O_3_ (8.03 wt% Cu, 7.95 wt% Zn)	Co-impregnation	230	1.25	GHSV = 1.70 L·g_cat._^−1^·h^−1^	92	1.25 ^e^	21.0		CH_3_OH conversion decreased from 92 to 87% after 125 h	[46]
7	38 wt% Cu/ZnO/Al_2_O_3_	Co-precipitation	225	1.3	WHSV_methanol_ = 6 h^−1^	67	0.07 ^d^	54.7	197	10% drop in CH_3_OH conversion after 40 h	[47]
8	Cu/ZnO/Al_2_O_3_ (Cu/Zn/Al (wt/wt) = 4/4/2.5)	Urea nitrate combustion	240	1.5	GHSV = 10.0 L·g_cat._^−1^·h^−1^	90	0			30% drop in CH_3_OH conversion after 90 h	[48]
9	20 wt% Cu/SiO_2_	Wet impregnation	280	1.5	WHSV = 2.55 h^−1^			91.6		Stable for 5 h	[49]
10	15 wt% Cu-MCM-41	One-pot hydrothermal method	250	3	GHSV = 2838 h^−1^	72.3	0.8 ^c^			No deactivation for 48 h	[50]
11	CeCuZn/CNTs (18 wt% Cu, 7 wt% Zn, 2 wt% Ce)	Microwave-assisted Polyol method	300	2	WHSV = 7.5 h^−1^	94.2	2.6 ^d^			7% drop in CH_3_OH conversion after 48 h	[51]
12	Cu/Ce-Cu(BDC) (10.1 wt% Cu, 2.9 wt% Ce)	Wet impregnation	250	1	WHSV = 9.2 h^−1^	99	2 ^d^			7% drop in CH_3_OH conversion after 32 h	[52]
13	8.3 wt% Cu/CeO_2_ nanorod	Hydrothermal method	260	1.2	GHSV_methanol_ = 800 h^−1^	100	2.4 ^d^	15.2			[53]
14	Cu/ZnO/CeO_2_/ Al_2_O_3_ (39 wt% CuO, 39 wt% ZnO, 11 wt% CeO_2_)	Sonochemical co-precipitation	200	1.5	GHSV = 10.0 L·g_cat._^−1^·h^−1^	100	0			No deactivation for 24 h	[54]
15	ZrO_2_/Cu (87.6 wt% CuO)	Oxalate sol-gel co-precipitation	200	1.0	WHSV = 10 h^−1^	32	0	52.8		No deactivation for 200 h	[55]
16	Cu/ZnO/ZrO_2_ (Cu/Zn/Zr = 36/47/17)	Co-precipitation	250	3	GHSV = 21.6 L·g_cat._^−1^·h^−1^	88.6	0	978	6970		[56]
17	7 wt% Cu/Ce_0.7_Zr_0.3_O_2_	Co-precipitation	240	1.5	WHSV = 27 h^−1^	23	0	87.8	503	No deactivation for 90 h	[57]
18	Cu/ZnO/CeO_2_-ZrO_2_ (CuO/ZnO/Support (wt/wt) = 45/20/35)	Co- precipitation	240	1.2	GHSV = 1200 h^−1^			22.8		No deactivation for 360 h	[58]
19	Cu/ZnO/CeO_2_/ ZrO_2_/SBA-15 (10 wt% Cu, 5 wt% ZnO, 10 wt% CeO_2_)	Incipient wetness impregnation	300	2	WHSV = 43.68 h^−1^	95.2	1.4 ^c^			12% drop in CH_3_OH conversion after 60 h	[59]
20	CuZnGaO_x_ (Cu:Zn:Ga = 43:47:10)	Co-precipitation	150	2	WHSV = 30 h^−1^	22.5	0	4.88	11.1		[60]
21	Cu20GaZn (10 wt% CuO, 20 wt% Ga)	Co-precipitation	200	1.3	WHSV_methanol_ = 6 h^−1^		0.2 ^d^	32.8	157	No deactivation for 24 h	[61]
22	CuZnGaZr (38.3 wt% CuO, 23.1 wt% ZrO_2_, 2.8 wt% Ga_2_O_3_)	Sol-gel	250	1	GHSV = 2200 h^−1^	42.9	0.3 ^f^	132		7% drop in CH_3_OH conversion after 44 h at 275 °C	[62]
23	10.6 wt% Cu/MgAl_2_O_4_	Wet impregnation	300	1	WHSV = 8.5 h^−1^	96	2.9 ^c^			4% drop in CH_3_OH conversion after 30 h at 200 °C	[63]
24	1.7 wt% Mg/Cu-Al spinel (Cu/Al = 1/3)	Incipient wetness impregnation	255	2.27	WHSV = 2.28 h^−1^	96.5	3.8 ^d^			No deactivation for 500 h	[64]
25	CuZnAl-5Mg (42.5 wt% Cu, 1.31 wt% Mg)	Co-precipitation	200	1	WHSV = 3.84 h^−1^	68.5	0.88 ^d^	47.8	180	18% drop in CH_3_OH conversion after 8 h at 350 °C	[65]
26	Cu_0.5_Ce_0.25_Mg_0.05_/Al (50 wt% Cu, 25 wt% Ce, 5 wt% Mg)	Co-precipitation	250	1.75	GHSV = 33.7 L·g_cat._^−1^·h^−1^	100	0.14~0.16 ^e^			No deactivation for 72 h	[66]
27	CuAl-0.1Fe (13 wt% Cu, 0.1 wt% Fe)	Co-precipitation	200	1.3	WHSV = 6 h^−1^		<0.1 ^d^	29.4	8.6		[67]
28	CuZnAl-0.1Fe (13 wt% Cu, 13 wt% Zn, 0.1 wt% Fe)	Co-precipitation	200	1.3	WHSV = 6 h^−1^		<0.1 ^d^	37.4	11.4		[67]
29	11.8 wt% Cu/CeO_2_	Colloidal solution combustion	250	1	WHSV_methanol_ = 7.2 h^−1^	66.3	0.8 ^d^	136	1274		[68]
30	6.8 wt% Cu-6.9 wt% CrO_x_/Al_2_O_3_	Co-impregnation	260	1.5	WHSV_methanol_ = 14.6 h^−1^	93.2	0.16 ^d^	317	3060	14% drop in H_2_ production rate after 50 h at 240 °C	[69]
31	10 wt% CuO/MgAl_2_O_4_	Wet impregnation	300	5	WHSV = 8.5 h^−1^	90.71	0.22 ^e^			No deactivation for 6 h at 350 °C	[70]
32	6.9 wt% Cu/Zn_0.4_Zr_0.6_O_x_	Wet impregnation	240	1.5	WHSV = 27 h^−1^			121	1253	12% drop in H_2_ production rate after 50 h at 240 °C	[71]
33	CuCo_2_O_4_	Co-precipitation	320	1.2	WHSV = 4.32 h^−1^	100	4.71 ^d^	66.7		35% drop in CH_3_OH conversion after 160 h	[72]

^a^ GHSV is the gas hourly space velocity, that is, the total volumetric flow rate of the gaseous reactants at standard temperature and pressure divided by the catalyst volume (h^−1^), or the total volumetric flow rate of the gaseous reactants at standard temperature and pressure divided by the catalyst mass (L·g_cat._^−1^·h^−1^). WHSV is the weight hourly space velocity, that is, the total mass flow rate of the liquid methanol solution divided by the catalyst mass (h^−1^). WHSV_methanol_ is the total mass flow rate of the liquid methanol divided by the catalyst mass (h^−1^). ^b^ Turnover frequency, ^c^ S_CO_ = $\frac{y_{CO}}{y_{CO\;}\;+\;y_{{CO}_{2}}+\;y_{{CH}_{4}}}\;\times \;$100%, ^d^ S_CO_ = $\frac{y_{CO}}{y_{CO\;}+\;y_{{CO}_{2}}}\;\times$ 100%, ^e^ S_CO_ = $\frac{y_{CO}}{y_{CO\;}+\;y_{{CO}_{2}}+\;y_{H_{2}}}\;\times \;$100%, ^f^ CO molar concentration in the exit stream.

**Table 3:** Some non-Cu-based catalysts for the steam reforming of methanol.

Entry	Catalysts	Preparation Method	Reaction Conditions			Catalyst Performance				Ref.
			Reaction Temperature (°C)	Feed Composition (H_2_O/CH_3_OH)	Space Velocity ^a^	CH_3_OH Conversion (%)	Selectivity to CO (S_CO_) (%)	Production Rate of H_2_ (μmol H_2_·g_cat._^−1^·s^−1^)	TOF ^b^ (h^−1^)	
1	1 wt% Pd/ZrO_2_-TiO_2_	Wet Impregnation	300	0.16	GHSV = 30,000 h^−1^	98	37 ^c^			[93]
2	1 wt% Pd-20 wt% Cu/ZnAl_2_O_4_	Wet impregnation	240		GHSV = 2400 h^−1^	100				[94]
3	3 wt% PdZn/ZnO	Impregnation	300	1	GHSV = 27.28 L·g_cat._^−1^·h^−1^	20~30				[95]
4	ZnPd/ZnO (0.94 wt% Pd)	Incipient impregnation	400	1.5	GHSV = 55.20 L·g_cat._^−1^·h^−1^	83				[96]
5	20 wt% Cu-4 wt% Pd/ZrO_2_	Sequential wet impregnation	220	1.5	GHSV = 2.460 L·g_cat._^−1^·h^−1^	63	5 ^d^	24.0		[97]
6	0.1 wt% Pd/ZnAl_2_O_4_	Incipient wet impregnation	250	1.1	WHSV = 6 h^−1^	35	3.0 ^d^	11.4	4351	[98]
7	3 wt% Pd/ZnO	Ethylene glycol reduction	400	1.2	GHSV = 36.6 L·g_cat._^−1^·h^−1^	94	0.5 ^d^	452		[99]
8	0.5 wt% Zn-0.5 wt% Pd/MoC	Incipient wetness impregnation	160	3	GHSV = 14.3 L·g_cat._^−1^·h^−1^	40.3	0.9 ^e^	19.1	1496	[100]
9	1 wt% Pd/In_2_O_3_/rod-shaped CeO_2_	Wet Impregnation	375	1.4	GHSV = 13,810 h^−1^	96	1.3 ^c^	69.4		[101]
10	0.89 wt% Pd-0.84 wt% Cu/ZnO	Incipient wetness impregnation	200	1.5	WHSV_methanol_ = 2.3 h^−1^	18.7	12.6 ^d^	10.1		[102]
11	Pt_1.6_Mo_98.4_C	Temperature-programmed reaction	200	1	GHSV = 9.0 L·g_cat._^−1^·h^−1^	100	3 ^c^			[103]
12	0.0125 wt% Pt/ZnO nanowires	Adsorption method	390	1.5	GHSV = 55.2 L·g_cat._^−1^·h^−1^	43		12.1	88,920	[104]
13	1 wt% Pt/3 wt% In_2_O_3_/CeO_2_	Incipient-wetness impregnation	325	1.4	GHSV = 12.87 L·g_cat._^−1^·h^−1^	98.7	2.6 ^d^	92.5	14,112	[105]
14	15 wt% Pt/30 wt% In_2_O_3_/Al_2_O_3_	Wet impregnation	350	1.4	GHSV = 99.0 L·g_cat._^−1^·h^−1^	95.9	1.0 ^c^			[106]
15	0.5 wt% Zn-2.0 wt% Pt/MoC	Temperature-programmed reaction	160	3	WHSV = 4.75 h^−1^	65.9		29.7	1098	[107]
16	0.3 wt% Pt-0.2 wt% K@Silicate-1	Ligand-protected hydrothermal	250	3	WHSV = 45 h^−1^			12.3	4201	[108]
17	Pt-CeCo (1.5 wt% Pt, 5.1 wt% Co)	Hydrothermal	200	3	WHSV_methanol_ = 5 h^−1^	30	7.3 ^c^	29.4	2120	[109]
18	1 wt% Pt/γ-Mo_2_N	Temperature-programmed reaction	200	3	WHSV_methanol_ = 12.87 h^−1^	28.4	<1	107	9837	[110]
19	0.013 wt% Pt-2 wt% La/γ-Mo_2_N	Temperature-programmed reaction	200	3	WHSV_methanol_ = 12.87 h^−1^	1.2	<5	7.5	41,038	[110]
20	0.26 wt% Pt-5 wt% La/γ-Mo_2_N	Temperature-programmed reaction	200	3	WHSV_methanol_ = 12.87 h^−1^	20.5	<1	86.5	23,374	[110]
21	4.7 wt% Ru/TiO_2_	Wet impregnation	300	2	WHSV = 1.8 h^−1^	98.9	5.4 ^e^			[111]
22	0.15 wt% Ru/porous CeO_2_ nanorods	Ascorbic acid-assisted reduction	350	3	GHSV = 49.2 L·g_cat._^−1^·h^−1^	25.6	2.2 ^d^	38.8	9493	[112]
23	0.15 wt% Rh/porous CeO_2_ nanorods	Ascorbic acid-assisted reduction	350	3	GHSV = 49.2 L·g_cat._^−1^·h^−1^	21	36 ^d^	27.8	6952	[112]
24	10 wt% Ni/γ-Al_2_O_3_-5H	Wet impregnation	450	2	GHSV = 19.273 L·g_cat._^−1^·h^−1^	100	5 ^c^			[113]
25	1 at.% Au-CeO_2_ nanorods	Deposition–precipitation	250	1.3	GHSV = 42.0 L·g_cat._^−1^·h^−1^	90	<3 ^d^	12.3		[114]
26	1 at.% Au-CeO_2_ nanorods	Deposition–precipitation	225	1.3	GHSV = 42.0 L·g_cat._^−1^·h^−1^	50				[115]
27	1 at.% Au/ZnO (polyhedra)	Deposition–precipitation	400	1.3	GHSV = 30.0 L·g_cat._^−1^·h^−1^	100		10.3		[116]
28	3 wt% Au/CeO_2_-Fe_2_O_3_	Deposition–precipitation	400	2	GHSV = 21.0 L·g_cat._^−1^·h^−1^	100	5 ^f^			[117]
29	3 wt% Au/Ce_0.75_Zr_0.25_O_2_	Co-precipitation	400	2	GHSV = 21.0 L·g_cat._^−1^·h^−1^	100	5 ^e^			[118]
30	3 wt% Au/CeO_2_–Fe_2_O_3_	Deposition–precipitation	350	2	GHSV = 21.0 L·g_cat._^−1^·h^−1^	100	2 ^f^			[119]
31	1 wt% Au/ZnZrO_x_	Anion-adsorption	350	1.3	GHSV = 34,000 h^−1^	100	0 ^d^	22.3	2484	[120]
32	CeO_x_/Au nanoparticles	Impregnation	300	10	GHSV = 350.0 L·g_cat._^−1^·h^−1^			66	216	[121]
33	3 wt% Au/CeO_2_	Deposition–precipitation	400	2 ^g^	GHSV = 30.0 L·g_cat._^−1^·h^−1^	100				[122]

^a^ GHSV is the gas hourly space velocity, that is, the total volumetric flow rate of the gaseous reactants at standard temperature and pressure divided by the catalyst volume (h^−1^), or the total volumetric flow rate of the gaseous reactants at standard temperature and pressure divided by the catalyst mass (L·g_cat._^−1^·h^−1^). WHSV is the weight hourly space velocity, that is, the total mass flow rate of the liquid methanol solution divided by the catalyst mass (h^−1^). WHSV_methanol_ is the total mass flow rate of the liquid methanol divided by the catalyst mass (h^−1^). ^b^ Turnover frequency, ^c^ S_CO_ = $\frac{y_{CO}}{y_{CO\;}+\;y_{{CO}_{2}}+\;y_{H_{2}}+\;y_{{CH}_{4}}}\;\times \;$100%, ^d^ S_CO_ = $\frac{y_{CO}}{y_{CO\;}+\;y_{{CO}_{2}}}\;\times \;$100%, ^e^ S_CO_ = $\frac{y_{CO}}{y_{CO\;}+\;y_{{CO}_{2}}+\;y_{H_{2}}}\;\times \;$100%, ^f^ CO molar concentration in the exit stream, ^g^ Molar ratio of O_2_:water:methanol = 0.6:2:1.

**Table 4:** Some noble metal catalysts for the aqueous-phase reforming of methanol.

Entry	Catalysts	Preparation Method	Reactor Type	Reaction Temperature (°C)	Feed Composition (H_2_O/CH_3_OH)	Production Rate of H_2_ (μmol H_2_·g_cat._^−1^·s^−1^)	TOF ^a^ (h^−1^)	Byproduct	Ref.
1	2 wt% Pt/α-MoC	Wet impregnation	Batch	190	3	130	4130	CO, CH_4_	[130]
2	0.2 wt% Pt/α-MoC	Wet impregnation	Batch	190	3	76.2	18,036	CO, CH_4_	[130]
3	2 wt% Pt/Al_2_O_3_	Wet impregnation	Batch	190	3	4.9	171	CO, CH_4_	[130]
4	2 wt% Pt/Al_2_O_3_	Incipient wetness impregnation	Batch	240	1	30.7	1077	CO	[131]
5	1 wt% Pt/NiAl_2_O_4_	Incipient wetness impregnation	Flow	210	16	7.32	662	CO, CH_4_	[132]
6	1 wt% Pt/γ-Al_2_O_3_	Incipient wetness impregnation	Flow	210	16	1.78	190	CO, CH_4_	[132]
7	0.50 wt% Pt/CeO_2_-H	UV-assisted impregnation	Batch	200	1		1290	CO, CH_4_	[133]
8	5 wt% Pt/Al_2_O_3_	A commercial catalyst	Flow	230	1.45	~8.33		CO, CH_4_	[134]
9	1.26 wt% Pt/Al_2_O_3_	Impregnation-reduction	Batch	150	20.2	0.302		CO, CH_4_	[135]
10	0.2 wt% Pt/γ-Mo_2_N(O_0.3_)	Incipient wetness impregnation	Batch	210	1	42.2	14,813	CO, CH_4_	[136]
11	0.05 wt% Pt@TiO_2_	In-situ photo deposition	Batch	190	3	1.89	2657		[137]
12	1.93 wt% Pt/CeO_2_-R (rod-shaped)	Photoreduction	Batch	250	16	6.77	1118	CO, CH_4_	[138]
13	2 wt% Pt/nanorod Al_2_O_3_	Wet impregnation	Batch	190	3	20.4	1276	CO, CH_4_	[139]
14	2.5 wt% Pt-0.5 wt% Fe/Al_2_O_3_	Incipient wetness impregnation	Batch	250	16	31.5		CO, CH_4_	[140]
15	1.5 wt% Pt–10 wt% Ni/sepiolite	Wet impregnation	Batch	240	57	26.9		CO, CH_4_	[141]
16	1 wt% Pt/NiAl_2_O_4_	Incipient wetness impregnation	Flow	210	16	7.32	662	CO, CH_4_	[132]
17	0.74 wt% Pt/LaNiO_x_-2	Deposition-precipitation	Flow	210	16	5.26		CO, CH_4_	[142]
18	3 wt% Pt/Na–ZrO_2_	Wet impregnation	Flow	260	16	7.20	162	CO, CH_4_	[143]
19	2 wt% Pt-1.29 wt% La/CeO_2_	Photochemical reduction	Batch	250	16	8.23		CO, CH_4_	[144]
20	1.72 wt% Pt-0.99 wt% MgO/CeO_2_	Sequential impregnation	Batch	250	16	3.81		CO, CH_4_	[145]
21	2 wt% PtMnK/AC	Initial wet impregnation	Batch	250	16	22.1		CO, CH_4_	[146]
22	1 wt% PtSn/C_3_N_4_	Solvothermal	Batch	200	3	165	11,581	CO, CH_4_	[147]
23	0.2 wt% PtSn/C_3_N_4_	Solvothermal	Batch	200	3	125	56,024	CO, CH_4_	[147]
24	1.4 wt% Pt/H_2_-In_2_O_3_	In-situ reduction	Batch	100	2.3	$7.69\times 10^{-2}$	96.9	CO, CH_4_	[148]
25	11 wt% Pt/Fe_5_C_2_@C	Photo-reduction deposition	Batch	200	1.2	38.9	238	CO, CH_4_	[149]
26	1.5 wt% Pt-1.0 wt% Au/MoS_2_-500H	Simultaneous wet impregnation	Batch	70	1	0.136	10.9	formate	[150]
27	2 wt% Pt/Ce_0.5_Mg_0.5_O_2_	Incipient wetness impregnation	Batch	250	16	29.4	2765	CO, CH_4_	[151]
28	Pt/Co_2_Al-700 (0.98 wt% Pt, 52.6 wt% Co, 11.9 wt% Al)	Incipient wetness impregnation	Batch	220	3	94.7	7992	CO	[152]
29	0.99 wt% Pt-0.45 wt% K/Al_2_O_3_	Impregnation	Batch	120	1	0.383	142	CO	[153]
30	0.36 wt% Pt_1_/porous CeO_2_ nanorods	Photo-assisted deposition	Batch	165	1	5.65	1103	CO	[154]
31	0.2 wt% Pt_1_/porous In_2_O_3_ nanocubes	Wet impregnation	Batch	180	1	5.48	1923	CO	[155]
32	Pt–Mo/TiO_2_	Conventional impregnation	Batch	81	20	0.140	13.7	CO	[156]
33	5 wt% Pt–2.46 wt% Mo/TiO_2_	Successive impregnation	Batch	84	20	0.258	12.1	CO	[157]
34	5 wt% Pt–2.59 wt% Ru/SiO_2_	Conventional impregnation	Batch	77-84	20	$3.42\times 10^{-2}$	2.01	CO, methyl formate	[158]
35	5 wt% Pt–2.59 wt% Ru/TiO_2_	Conventional impregnation	Batch	77-84	20	$5.72\times 10^{-2}$	2.30	CO	[159]

^a^ Turnover frequency.

**Table 5:** Some non-noble metal catalysts for the aqueous-phase reforming of methanol.

Entry	Catalysts	Preparation Method	Reactor Type	Reaction Temperature (°C)	Feed Composition (H_2_O/CH_3_OH)	Production Rate of H_2_ (μmol H_2_·g_cat._^−1^·s^−1^)	TOF ^a^ (h^−1^)	Byproduct	Ref.
1	13 wt% Ni-1.3 wt% Ce/γ-Al_2_O_3_	Wet impregnation	Flow	230	34	1.41		CH_4_, C_2_H_6_	[160]
2	Ni_4_CSZ (5 wt% Ni, 1.20 wt% Ca, 60 wt% Zr)	Incipient wetness impregnation	Flow	230	34	3.37	216	CO, CH_4_	[161]
3	NiCe_4_CSZ (5.9 wt% Ni, 23 wt% Ce, 1.8 wt% Ca, 47.6 wt% Zr)	Incipient wetness impregnation	Flow	230	34	3.01	202	CO, CH_4_	[161]
4	10 wt% Ni/25 wt% CeO_2_-ZrO_2_	Incipient wetness impregnation	Flow	230	34	42.2	240	CO, CH_4_	[162]
5	La-promoted NiMgAl hydrotalcite (38 wt% Ni, 5.4 wt% La)	Co-precipitation	Batch	230	16	9.03		CO, CH_4_	[163]
6	Ni_3_Mg_1_-Mixed metal oxide (58 wt% Ni, Ni/Mg/Al = 3.5/1.1/1)	Co-precipitation	Batch	230	3	133.1	2376	CO, CH_4_	[164]
7	2 wt% Ni/α-MoC	Incipient wetness impregnation	Batch	240	1	171	1805	CO	[131]
8	2 wt% Ni/Mo_x_C-3	Wet impregnation	Batch	240	2	9.20	97.2	CO, CH_4_	[165]
9	40 wt% Ni@N-doped carbon	Sol-gel	Batch	240	3	70.7		CO, CH_4_	[166]
10	40 wt% Ni@N-doped carbon + 0.86 M KOH	Sol-gel	Batch	240	3	406		CO, CH_4_	[166]
11	Ni_0.1_/HC-N_1.5-_S_1_ (1.9 wt% Ni)	Hydrothermal carbonization–pyrolysis	Batch	250	9	95.8	5370	CO, CH_4_	[167]
12	C-modified NiMgAl (37.41 wt% Ni)	Co-precipitation	Batch	230	16	12.1		CO, CH_4_	[168]
13	55 wt% Cu/ZnO/CeO_2_	Co-precipitation	Batch	210	2	58.4	23.8	CO, CH_4_	[169]
14	Ga_2_O_3_-modified Cu−ZnO (38.9 wt% Cu, 26.6 wt% Zn, 11.9 wt% Ga)	Citric acid-assisted sol-gel	Batch	210	1	101	446	CO, CH_4_	[170]
15	68 wt% Cu@N-doped graphitic carbon	Sol-gel	Batch	210	3	91.9		CO, CH_4_	[171]
16	Cu-CuO_X_/C-700	Solvothermal	Batch	210	1	33.7		CO, CH_4_	[172]
17	Cu@Citric acid-Valine	Citric acid-assisted sol-gel	Batch	180	1	27.0	14	CO, CH_4_	[173]
18	45 wt% Cu@N-doped carbon-200	Sol-gel	Batch	210	3	34.0		CO	[174]
19	Cu-CeO_2_@Polyvinylpyrrolidone (40 wt% Cu, 0.27 wt% Ce)	Sol-gel	Batch	180	1	32.6		CO, CH_4_	[175]
20	52.9 wt% Cu@SS-Arg	Sol-gel	Batch	210	1	31.2		CO, CH_4_	[176]
21	0.67Cu/Lysine-Citric acid-400	Sol-gel	Batch	210	1	51.2		Formate, formic acid	[177]
22	Cu/Cu_2_O/CuN_3_@NC (67.6 wt% Cu)	Polyvinylpyrrolidone-assisted sol-gel	Batch	210	1	140		CO, CH_4_	[178]
23	35 wt% Cu@CS_19_/G_1_-300 (CS: Chitosan, G: Glucose)	Sol-gel	Batch	210	3	38.6		CO	[179]
24	53 wt% Cu-SP/Al_2_O_3_–ZnO (SP: sesbania powder)	Sesbania powder-assisted sol-gel	Batch	210	3	64.2	686	CO, CH_4_	[180]
25	ZnO/Ni-8Cu/Al_2_O_3_ (10 wt% Ni, 8 wt% Cu, 15 wt% ZnO)	Co-impregnation	Batch	250	16	6.04		CO, CH_4_	[181]
26	Ni/Cu/Cu_2_O@Citric acid	Sol-gel	Batch	240	1	136		CO, CH_4_	[182]
27	Fe–Cu@(N)G (8.2 wt% Cu, 2.5 wt% Fe, 4.2 wt% N, 69.7 wt% C)	Impregnation-pyrolysis	Batch	190	1	153	317	CH_4_	[183]

^a^ Turnover frequency.

**Table 6:** Some catalysts for low-temperature methanol dehydrogenation.

Entry	Catalysts			Amount of Catalyst	Additives	Temperature (°C)	Feed Composition (CH_3_OH:H_2_O)	TOF ^a^ (h^−1^)	Comments	Ref.
1 ^b^	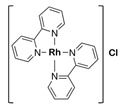 [Rh(2,2′-bpy)_2_]Cl			10^−3^ mol/L	1.0 M NaOH	120	1:0	7		[190]
2	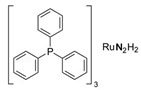 [RuH_2_(N_2_)(PPh_3_)_3_]			1~5 × 10^−4^ mol/L	1.0 M NaOH	150	1:0	6.4		[191]
3	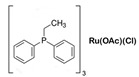 [Ru(OAc)(Cl)(PEtPh_2_)_3_]			0.25 × 10^−3^ mol/L		66	1:0	0.60		[192]
4	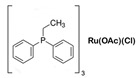 [Ru(OAc)(Cl)(PEtPh_2_)_3_]			0.25 × 10^−3^ mol/L	[Acetic acid]/[Ru] = 2	66	1:0	0.96		[193]
5	Ruthenium trichloride hydrate			5 × 10^−3^ mol/L	20 wt.% NaOCH_3_	79	1:0	1.68	Byproducts: NaCOOH, dimethoxymethane	[194]
6	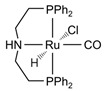 [RuHCl(CO)(HN(C_2_H_4_PPh_2_)_2_)]			19 ppm	8.0 M KOH	91	9:1	1023		[195]
7	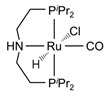 [RuHCl(CO)(HN(C_2_H_4_P*i*Pr_2_)_2_)]			19 ppm	8.0 M KOH	91	9:1	2276		[195]
8	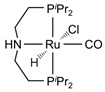 [RuHCl(CO)(HN(C_2_H_4_P*i*Pr_2_)_2_)]			1.6 ppm	8.0 M KOH	95	1:0	4719		[195]
9	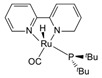			0.025 mol%	KOH, toluene	100–105	10:1	43	Yield to H_2_: 77%, no decrease in catalytic activity for ∼1 month	[196]
10 ^c^	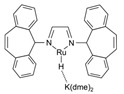 [K(dme)_2_][Ru(H)(trop_2_dad)]			0.5 mol%	THF	90	1:1		~80% conversion after 10 h with 1 g of MeOH	[197]
11	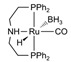	+	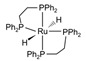	5 μmol + 5 μmol	triglyme	93.5	9:1	93		[198]
12	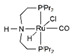	+	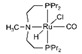	8.56~9.62 μmol	triglyme	92.5	9:1	194		[199]
13	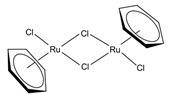			0.625 mol%	KOH and [Ru]:[2-hydroxypyridine] = 1:2	130	1:1	49	Yield to H_2_: 50%	[200]
14	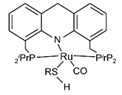				Neutral condition	150	9:1	643	Yield to H_2_: 96%, Turnover number (23 d) = 130,000	[201]
15 ^d^	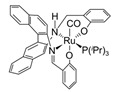 Ru(salbinapht)(CO)(P*i-*Pr_3_)			12 μmol	8.0 M KOH, dioxane	82	9:1	55		[202]
16 ^e^	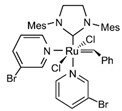			5 μmol	8 M KOH	88	9:1	139	Turnover number (72 h) = 11,424, [CO] = 0.8 × 10^−4^ ppm	[203]
17 ^e^	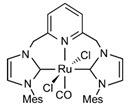			4 μmol	8 M KOH	94	9:1	248	Yield to H_2_: 7.1% and yield to HCOOH: 7.1%	[204]
18	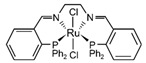			4 μmol	8 M KOH, triglyme	120	8:2	1066		[205]
19	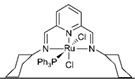			0.04 mol%	KO^t^Bu	100	4.5:1		Yield to H_2_: 24% and yield to formic acid: 24%	[206]
20	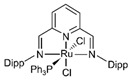			3 μmol	8 M KOH	120	9:1	193		[207]
21 ^f^	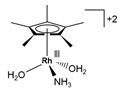 [Cp*Rh(NH_3_)(H_2_O)_2_]^3+^			3.4 μmol	Buffer solution (pH = 6)	70	9:1	83.2		[208]
22 ^g^	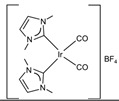			3 μmol	Basic condition (20 mmol KOH)	91	1:0	112	Turnover number (24 h) = 3612, Yield to H_2_: 81%	[209]
22	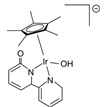			0.5 mol%		<100	1:4		Yield to H_2_: 10%	[210]
23	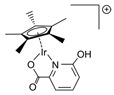			0.5 mol%	0.50 mol% NaOH	<100	1:4	70	Yield to H_2_: 84%	[210]
24	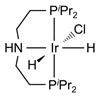			4.18 μmol	0.5 M KOH	70	9:1	326		[211]
25	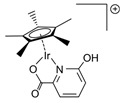			0.5 mM	1.58 mM Na_2_CO_3_	78	1:0	377		[212]
26	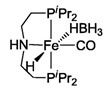 [FeH(BH_4_)(CO)(HN(C_2_H_4_P^i^Pr_2_)_2_)]			4.18 μmol	8.0 M KOH	91	9:1	702	Turnover number (46 h) = 6270	[213]
27	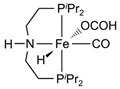			0.01 mol%	LiBF_4_ (10 mol %), ethyl acetate	77	4:1	577	Turnover number = 30,000, Yield to H_2_: >99%	[215]
28	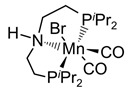			1.68 mM	8.0 M KOH	92	9:1	11	Yield to H_2_: 7%, Turnover number (900 h) = 20,000	[214]

^a^ Turnover frequency, ^b^ 2,2′-bpy = 2,2′-bipyridine, ^c^ dme = 1,4-diaminoethane, trop2dad = 1,4-bis(5H-dibenzo[a,d]cyclohepten-5-yl)-1,4-diazabuta-1,3-diene, ^d^ salbinapht = 2-[({2′-[(2-hydroxybenzyl)amino]-[1,1′-binaphthalen]-2-yl}imino)methyl]phenolato, ^e^ Mes = 2,4,6-Me_3_C_6_H_2_, ^f^ Dipp = diisopropylphenyl, ^g^ Cp = cyclopentadienyl.

## Data Availability

No new data were created or analyzed in this study. Data sharing is not applicable to this article.
